# Reading

**Published:** 1880-04

**Authors:** 


### READING.

What a beautiful, and useful, and pleasure-producing employment is it, to read in the family-circle! Let there be stated times for the exercise. Let every one select gems of thought—elegant literature, scientific truth, rare and interesting information, and bring it forward for the general good. A good article is as quickly read as a poor one. A single paragraph, of extraordinary force and beauty, is of more value than whole volumes of fair common-place reading. One scientific truth, clearly brought out, will furnish matter for useful conversation, and reflection, and profitable application, for many years to come.

Let the individual members be appointed each to seek out and present some specific needed information. Appoint each one to lecture on a given subject. Give to one, for instance, "printing," as a theme, with instructions to acquire and present to the family-circle, in a compact form, information on the whole subject, embracing the etymology of the word *to print;* the origin of the art—its progress—its present extent in the world—its connection with other discoveries and improvements, as paper and ink manufacture, and printing-presses, and types, stereotyping and electrotyping, and their present and prospective influence upon the world. Give to another, Africa, embracing its physical geography, and races, and languages, with commerce, and productions, and travels, and colonization, and present and prospective civilization—slavery, soil, climate, and indeed everything that can awaken an intelligent interest. Let another prepare a lecture on chemical science; another, on the beautiful; another, on the vast; and another, on the minute. Give time for preparation by reading and inquiry. Allow months or weeks—if needs be, a year. To this lyceum work, manufacture may be added; and every member may be required to produce something distinguished for elegance or use. Let song, and utterance, and the vernacular tongue, be cultivated. Let sketches of excursions be preserved, and scraps of great value be clipped from periodical literature, and preserved. These are mere hints. Carry the purpose of improvement out in all directions. Make the family a university.

---

Parental Responsibility.—The father molds the head; the mother, the heart; the father appeals to the understanding; the mother, to the affections; the father prepares for time; the mother, for eternity. Happy the children who heed the wise teachings of both.

